# The loss of ATP2C1 impairs the DNA damage response and induces altered skin homeostasis: Consequences for epidermal biology in Hailey-Hailey disease

**DOI:** 10.1038/srep31567

**Published:** 2016-08-16

**Authors:** Samantha Cialfi, Loredana Le Pera, Carlo De Blasio, Germano Mariano, Rocco Palermo, Azzurra Zonfrilli, Daniela Uccelletti, Claudio Palleschi, Gianfranco Biolcati, Luca Barbieri, Isabella Screpanti, Claudio Talora

**Affiliations:** 1 Department of Molecular Medicine, Sapienza University of Rome, Rome, Italy; 2 Center for Life Nano Science@Sapienza, Istituto Italiano di Tecnologia, Rome, Italy; 3 Department of Biology and Biotechnology “C. Darwin”; Sapienza University of Rome, Rome, Italy; 4 Porphyria Center, San Gallicano Institute IRCCS, Rome, Italy; 5 Istituto Pasteur Italia, Fondazione Cenci-Bolognetti, Italy.

## Abstract

Mutation of the Golgi Ca^2+^-ATPase *ATP2C1* is associated with deregulated calcium homeostasis and altered skin function. *ATP2C1* mutations have been identified as having a causative role in Hailey-Hailey disease, an autosomal-dominant skin disorder. Here, we identified *ATP2C1* as a crucial regulator of epidermal homeostasis through the regulation of oxidative stress. Upon *ATP2C1* inactivation, oxidative stress and Notch1 activation were increased in cultured human keratinocytes. Using RNA-seq experiments, we found that the DNA damage response (DDR) was consistently down-regulated in keratinocytes derived from the lesions of patients with Hailey-Hailey disease. Although oxidative stress activates the DDR, *ATP2C1* inactivation down-regulates DDR gene expression. We showed that the DDR response was a major target of oxidative stress-induced Notch1 activation. Here, we show that this activation is functionally important because early Notch1 activation in keratinocytes induces keratinocyte differentiation and represses the DDR. These results indicate that an *ATP2C1/NOTCH1* axis might be critical for keratinocyte function and cutaneous homeostasis, suggesting a plausible model for the pathological features of Hailey-Hailey disease.

Epidermal homeostasis depends on the continuous balance between the proliferative and differentiation potential of keratinocytes, which exit the cell cycle and differentiate as they move outward from the proliferative basal layer through the suprabasal layers^1^,^2^,^3^. Several signaling pathways regulate the morphological and functional changes associated with epidermal differentiation. These signals and changes are important for epidermal barrier function and responses to external environmental insults. In keratinocytes, complex and fine-tuned calcium signaling gives rise to a number of signaling pathways involved in differentiation. A crucial aspect of calcium signaling is the maintenance of the cytoplasmic calcium level by an array of calcium pumps and exchangers located on the surfaces of intracellular membranes^4^,^5^. The orthologue of a yeast gene encoding a calcium ATPase, *ATP2C1*, is thought to play a critical role in the pathogenesis of Hailey-Hailey disease (HHD)^6^,^7^,^8^,^9^. HHD is a rare autosomal-dominant inherited disease with high penetrance. It is characterized by suprabasal cell separation (acantholysis) of the epidermis. Although HHD is a rare disorder, it is a very good model by which to study the different key regulatory systems of the skin, including keratinocyte proliferation, differentiation and adhesion. Indeed, the information obtained for HHD can be translated into other diseases with similar underlying pathogenesis. We showed an increase in oxidative stress in keratinocytes derived from cutaneous lesions of HHD patients^10^,^11^,^12^. Interestingly, we showed that Notch1 expression is negatively regulated by *ATP2C1* deficiency-mediated reactive oxygen species (ROS) induction in keratinocytes, both *ex vivo* and *in vitro*^10^,^11^,^12^. Perhaps the best evidence underscoring the importance of ROS and *NOTCH1* is the recently established role of this axis in the process of keratinocyte differentiation^13^. ROS generated by the mitochondria are important regulators of epidermal differentiation. A failure to generate mitochondria-derived ROS impaired epidermal differentiation by preventing the transmission of Notch signals that are essential for epidermal differentiation^13^. Although terminal differentiation of keratinocytes includes cell death, it has become clear during the past decade that this pathway also employs pro-survival mechanisms^14^. Recent studies have determined that stem cells can undergo cell differentiation upon DNA damage in an *ATM (*protein kinase ataxia-telangiectasia mutated)-dependent manner^15^. *ATM,* best known for its role as an apical activator of the DNA damage response (DDR), mobilizes and orchestrates one of the most extensive signaling networks in response to the induction of DNA damage and directly or indirectly modifies a broad range of targets^16^. Oxidative DNA damage caused by ROS generated during metabolism makes significant contributions to genomic instability, carcinogenesis and cellular aging^16^,^17^,^18^. *ATM* has also been directly implicated in the regulation of oxidative stress, and several reports have indicated that oxidative stress was not properly controlled in cells from patients with ataxia-telangiectasia and in tissues of *ATM*-deficient mice^16^,^17^,^18^. Notably, genotoxic stress induces an increase in DNA damage that blocks transcription and might down-regulate DNA repair itself. Notch is a direct negative regulator of the DDR that counteracts ATM signaling^19^. Interestingly, the DDR is activated upon oxidative DNA damage that results from ROS. It has been shown that MYC hyperactivity causes the accumulation of DNA damage and terminal squamous differentiation^20^.

Much about the molecular mechanisms regulating the complex interactions among resident skin cells and the additional extrinsic signals involved in HHD manifestation remains unknown. In this study, RNA-seq was used to identify the differentially expressed genes in lesioned (LS) and non-lesioned (NL) HHD skin samples from the same individual. Three pairs of LS and NL skin biopsies were taken and analyzed. RNA-seq data analysis identified a substantial number of differentially expressed genes in lesioned skin. These changes in gene expression were clearly shown by the down-regulation of genes involved in the DDR and the up-regulation of genes related to the inflammatory response. Dysregulation of inflammatory genes contributes to altered cutaneous homeostasis and to the pathogenesis of inflammatory skin diseases^21^. Our data raise the possibility that keratinocyte-intrinsic pathways have key roles in regulating immune homeostasis and inflammation in HHD-lesioned skin. We have shown that the differentiation of HHD-derived keratinocytes is impaired, suggesting that ROS may play an early, causal role in the altered HHD-derived keratinocyte differentiation^10^,^11^,^12^. Our hypothesis is that the disturbance of calcium homeostasis in Hailey-Hailey keratinocytes promotes skin lesions by causing DNA damage and that Notch1 might mediate this response. This signaling can lead to a level of DNA damage that overwhelms the defenses of the keratinocyte cells and causes their death. Our results may contribute to a better characterization of a subset of human skin diseases and guide new therapeutic treatments.

## Results

### Differential gene expression between the lesioned and non-lesioned skin of individuals with Hailey-Hailey disease

**Figure 1:**
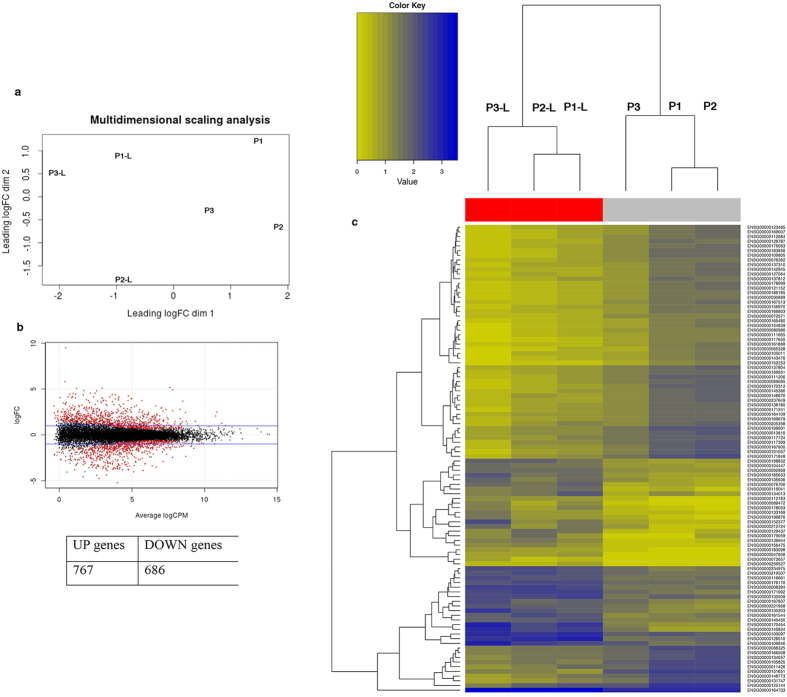
(**a**) Multidimensional scaling (MDS) plot generated using the edgeR package. The distance between the sample labels indicates similarity. Lesioned samples are named P1-L through P3-L. Non-lesioned samples are named P1 through P3. Dimension 1 separates the lesioned samples from the non-lesioned samples, whereas dimension 2 roughly corresponds to the patient number. This result confirms the paired nature of the samples. (**b**) Plot showing the relationship between the log2-fold change versus the average log2-counts-per-million (CPM) of the genes; the horizontal lines indicate a fold change of two. Red font was used to indicate the differentially expressed genes at an FDR of 0.05; in the table the corresponding numbers of up- and down-regulated genes. (**c**) Unsupervised hierarchical clustering of the expression profiles of the non-lesion- and lesion-derived keratinocyte samples from HHD patients was performed using the top 100 differentially expressed genes. The color scale from yellow to blue corresponds to their expression values. The Ensembl gene names are labeled on the dendrogram. Lesion-derived samples are named P1-L through P3-L. Control samples are named P1 through P3.

**Figure 2:**
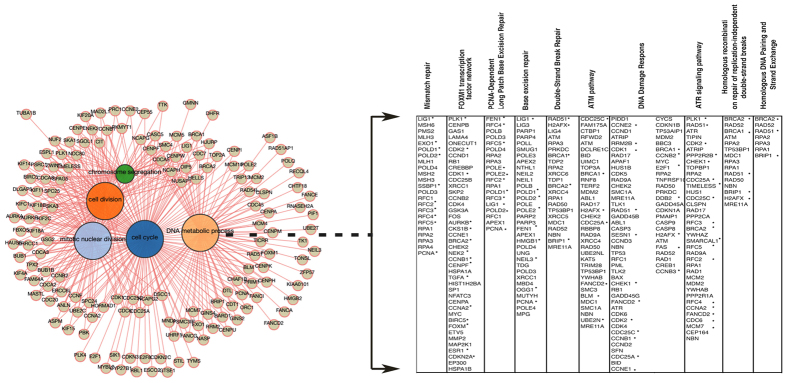
Network graphic output of the BioMart enrichment tool, which reports the Gene Ontology (GO) enrichment analysis performed using 686 down-regulated genes from the keratinocytes derived from the HHD lesions (indicated by * in the table). The functional annotations were further analyzed using the Consensus Path Database (cpdb.molgen.mpg.de).

HHD is a chronic, rare inherited disease of the skin characterized by red scaly areas that can be painful and itchy and can lead to superficial blisters and eroded areas of the skin. The disease often has a remission and recurrence pattern, which may be constant in some patients. HHD is associated with the loss of a single copy of *ATP2C1,* a gene that is likely essential in humans, as more severe phenotypes are found in patients who suffer clonal loss of both copies of the gene^22^. Consistently, mice embryos homozygous for null mutations in *ATP2C1* die due to defects in neural tube closure, whereas heterozygotes show susceptibility to squamous cell tumors, a phenotype that is rarely observed in humans with HHD (ref. 23,24 and our personal observations). ATP2C1 is localized in the Golgi, where it transports both Ca^2+^ and Mn^2+^ and plays important roles in the homeostasis of both molecules^4^,^25^,^26^,^27^. It remains an enigma why individuals with defects in the *ATP2C1* gene develop a skin-specific disease, highlighting the need to elucidate how genetics might contribute to the tissue specificity of HHD. Accordingly, we performed whole-genome expression profiling using RNA-seq data from lesioned (P1-L, P2-L, and P3-L) and unaffected skin (P1, P2, and P3) of three individuals with HHD to identify the differentially expressed genes. For each sample, an average of ~50 million 100-bp reads was generated. The sequenced reads were mapped to the human reference genome (hg19), with an average of 46.5 million mapped reads per sample. An average of ~85% of all the aligned reads were uniquely mapped on the genome and were considered for read counting, with respect to the Ensembl transcriptome annotation^28^. Normalization and the analysis of differential gene expression were performed using the EdgeR package^29^,^30^. The multidimensional scaling analysis supported the partition of the non-lesioned *vs* lesioned samples into two groups (Fig. 1a). A total of 1,453 genes were significantly differentially expressed between the normal and lesion-derived keratinocytes at a false discovery rate (FDR) < 0.05. The expression of 686 genes was down-regulated and the expression of 767 genes was up-regulated in the lesions compared with the unaffected skin controls (Fig. 1b). Hierarchical clustering of gene expression clearly showed separation between the keratinocytes from the non-lesioned and lesioned keratinocytes; the corresponding heatmap of the top 100 differentially expressed genes is shown in Fig. 1c. A functional annotation was performed to identify the biological processes that were significantly enriched in the differentially expressed genes. An enrichment map was generated to visualize the functional clusters of the enriched biological processes, including cell cycle, DDR and metabolic process (Fig. 2). DNA damage/DNA repair was the main cluster in the enrichment map, and the genes involved in the process were predominantly down-regulated in the lesioned skin of HHD patients compared with the unaffected controls (Fig. 2 and Supplementary Fig. 1). We found that the HHD lesion-derived keratinocytes had a higher level of oxidative stress than the non-lesion-derived keratinocytes^10^,^11^,^12^. Oxidative stress can result in oxidative DNA damage; if the DNA-damage remains unrepaired, the cells may enter cellular senescence or programmed cell death^31^,^32^,^33^. Interestingly, we found that HHD lesion-derived keratinocytes were hypoproliferative compared to the non-lesion-derived keratinocytes^11^. Therefore, it is possible that persistent DDR repression is causally associated with HHD lesion manifestation. We performed whole-exome sequencing of two lesion-derived keratinocytes using human all-exon targeted capture (Agilent) followed by massively parallel sequencing (Illumina) to address this important question. The mean sequencing coverage across the targeted bases was 150X, and 85% of the bases had more than 100X coverage. After some variant filtering steps (see Methods), we identified a total of 1,520 missense mutations that were shared between the lesions derived from the two patients (Supplementary Fig. 2), most of which (~400) were novel non-synonymous variants that had not already been annotated in dbSNP (Build 142)^34^. The mutation burden we identified in the HHD lesions was greater than those of most other common genetic diseases, as previously described^35^; for example, the mutation burden was comparable to the burden observed in Seckel Syndrome, in which the functional loss of the centrosomal protein *CEP152* results in an impairment of DDR pathways^35^,^36^. We also identified 45 null mutations that were common to both lesions (Supplementary Fig. 2a,b and Supplementary Fig. 3a,b). Nonsense mutations were identified in *IRS1* (insulin receptor substrate), which is essential for skin formation and development^37^. The lesions harbored an additional nonsense mutation that disrupted *PRIM2*, a DNA primase that plays a key role in DNA replication and in the DDR^38^. We also identified nonsense mutations targeting the *CROCC* (ciliary rootlet coiled-coil or Rootletin) gene, which forms centriole-associated filaments and functions in centrosome cohesion^39^. Conceivably, these lesions harbor mutations in genes whose loss would be deleterious to skin cells. Overall, the results show that the DDR is strongly repressed in HHD lesions. Consequently, the keratinocytes derived from the HHD lesions tend to be lost, likely as a result of the sequential acquisition of mutations in genes involved in the cellular stress response.

### ATP2C1 loss in keratinocytes is associated with Notch1 activation and ATM down-regulation

**Figure 3:**
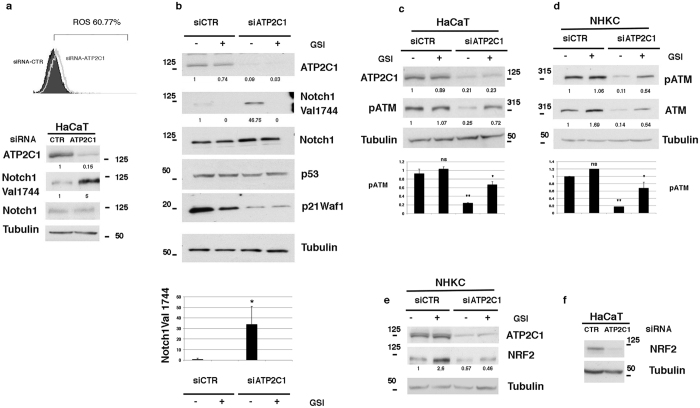
Decreased ATM levels by knockdown of ATP2C1 expression. (**a**) In the upper panel, the HaCaT keratinocyte-derived cell line was transfected with either siRNA-CTR or siRNA-ATP2C1, and the cells were analyzed by flow cytometry. The percentage of positive cells is also shown. Overlay of the FACS profile of the siRNA-CTR-transfected keratinocytes (filled histogram) onto the profile of the siRNA-ATP2C1-transfected keratinocytes (empty histogram). In the lower panel, the HaCaT cells were treated as in the upper panel and were analyzed by western blotting for ATP2C1, Notch1, and Notch1Val1744. Tubulin blots are shown as a control for equal loading. (**b**) Primary human keratinocytes were transfected with either siRNAs targeting ATP2C1 or a scrambled siRNA control. The cells were analyzed by western blotting for ATP2C1 and the indicated proteins at 48 h after transfection. Twenty-four hrs after transfection, the cells were treated with GSI (10 μΜ), incubated for an additional 24 h, and analyzed by immunoblotting with the indicated antibodies. Each data point in the graph represents the mean ± SEM of three independent experiments. *P < *0.05*; lanes 1 *vs* lane 3. (**c**,**d**) Both NHKCs and HaCaT cells were transfected with the control (siRNA-CTR) or ATP2C1-specific siRNAs; 24 h later, the cells were treated with GSI (10 μΜ) for 24 h and analyzed by immunoblotting with the indicated antibodies. In panels b and d, the same cell extracts were analyzed on separated gels. In panels c and d, each data point in the graph represents the mean ± SEM of three independent experiments. ns lane 1 *vs* lane 2; **P < *0.005*, lane 1 *vs* lane 3; *P < *0.05*, lane 3 *vs* lane 4; (**e**) NHKCs were treated as in panel b and analyzed by western blotting for ATP2C1 and NRF2. Tubulin is shown as a control for equal loading. (**F**) HaCaT cells were treated as in panel a and were analyzed by Western blotting for NRF2. Tubulin is shown as a control for equal loading.

Because HHD is associated with *ATP2C1* loss, we examined the consequences of *ATP2C1* inactivation in the DDR response in human primary keratinocytes and keratinocyte-derived cell lines. HHD lesion-derived keratinocytes are characterized by increased oxidative stress and decreased expression levels of both Notch1 and NRF2^11^. Interestingly, Nrf2 activation directly regulates DDR gene transcription^40^; thus, the loss of Nrf2 in lesioned HHD skin may play a role in the down-regulation of the transcription of DDR genes. Therefore, we first confirmed that *ATP2C1* loss increased oxidative stress (Fig. 3a). The percentage of DFCA-positive cells in siATP2C1 cells reached 60% at 48 hrs after transfection, whereas only 15% of the siRNA-CTR control cells were DFCA-positive. Unexpectedly, inhibition of *ATP2C1* expression in keratinocytes resulted in increased levels of activated Notch1 in both HaCaT cells and primary keratinocytes (Fig. 3a lower panel and 3b). Conversely, NRF2 expression was down-regulated in both HaCaT cells and primary keratinocytes, as previously shown in primary keratinocytes derived from the HHD lesions (Fig. 3e,f). Recently, it has been shown that Notch inactivation of ATM kinase activity represents an evolutionarily conserved mechanism that impairs the DDR response^19^. Notch1 binds to ATM and directly inhibits its kinase activity. Therefore, we tested whether Notch1 signaling is involved in the control of ATM/DDR in keratinocytes. Interestingly, the level of the activated Notch1 protein was increased upon transfection with an siRNA for the *ATP2C1* gene (Fig. 3b). Strikingly, cells expressing activated Notch1 showed reductions in both phosphorylated and total ATM compared to the control cells (Fig. 3b–d). However, when we inhibited Notch1 activation by adding a γ-secretase inhibitor (GSI), the ATM levels were no longer reduced in the siATP2C1-treated cells (Fig. 3b–d).

### Notch1 down-regulated ATM during keratinocyte differentiation

**Figure 4:**
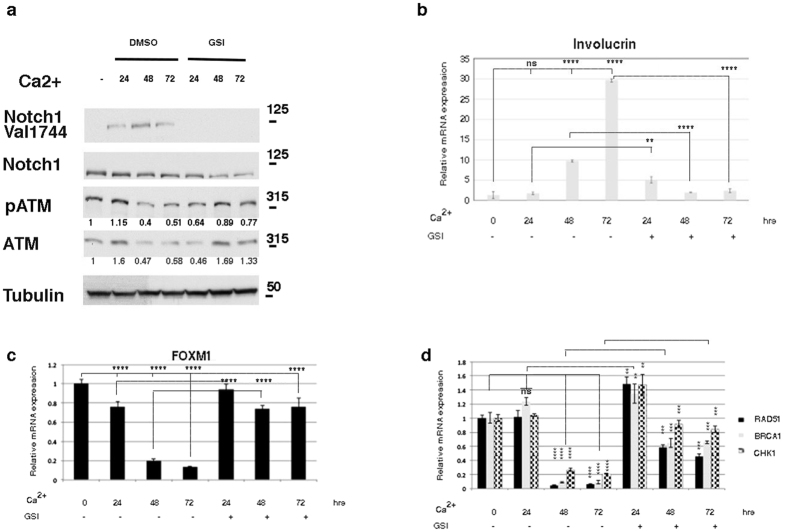
Calcium down-regulates the DNA damage response. (**a**) Cell extracts were prepared from undifferentiated NHKC cells and cells that were differentiated with high calcium concentrations for 24, 48 and 72 hours. Calcium-induced differentiation was performed in the presence of dimethyl sulfoxide (DMSO) as a vehicle control or GSI (10 μΜ). Lysates were analyzed with the indicated antibodies. For the ATM analysis, the membrane was first analyzed with a pATM antibody and was then re-probed with an antibody against the total ATM protein (**b–d**) NHKCs were treated as in (**a**) and expression levels of the differentiation marker Involucrin and genes involved in the DDR pathways were determined by qRT-PCR. All graphs are representative of three independent experiments, and the values are expressed as the means ± SD of experiments conducted in triplicate. The significance of the differences was calculated using two-tailed Student’s *t* test, with the vehicle-treated sample as the reference, and one-way ANOVA by comparing the mean of each indicated column. In panel b, ****P < *0.0001* and **P = *0.002*. In panel c, ****P < *0.0001*. In panel d, ****P < 0.0001, **P = 0.001 and ***P = *0.0001.*

The Notch pathway plays an important role in regulating epidermal differentiation^41^,^42^,^43^,^44^. Genetic ablation or activation of the pathway reveals that Notch signaling promotes the differentiation of the interfollicular epidermis. Using a calcium-induced keratinocyte differentiation model, we investigated the interaction between Notch1/ATM and DDR signaling during keratinocyte differentiation. Ca^2+^ treatment induces cell cycle arrest and morphological changes typical of keratinocyte differentiation in cultured cells (Supplementary Fig. 6a,b). Upon further investigation by real-time PCR, the analysis revealed that calcium-induced keratinocyte differentiation specifically increased expression of specific suprabasal markers and Notch1 signaling, changes that were inhibited by GSI treatment (Fig. 4a,b). We analyzed the mRNA expression levels of the DDR factors FoxM1, RAD51, BRCA1 and CHK1 by quantitative RT-PCR (qRT-PCR) in Ca^2+^-differentiated keratinocytes to determine whether DDR signaling is modulated by keratinocyte differentiation. We found that the transcription levels of all these genes were strongly down-regulated in the Ca^2+^-treated keratinocytes (Fig. 4c,d). We next analyzed the expression levels of all investigated genes upon treatment with a Notch inhibitor. As shown in Fig. 4c,d, the mRNA levels of DDR genes were slightly or unaffected by GSI treatment. Notably, the same expression profile was observed for ATM protein expression (Fig. 4a). Our analysis of the DDR factors provided evidence that DDR gene expression was significantly decreased in keratinocytes after calcium treatment. The Notch inhibitor blocked the calcium-induced repression of the DDR genes and the induction of keratinocyte differentiation. These data indicate that calcium decreases ATM/DDR by increasing Notch1 signaling, indicating that the inactivation of both ATM and DDRs might also be involved in driving the cell toward differentiation. However, although ATM down-regulation was correlated with the activation of Notch1 signaling during calcium-induced keratinocyte differentiation, ATM inhibition was not sufficient to clearly promote keratinocyte differentiation. Thus, although ATM inhibition results in DDR down-regulation (Supplementary Fig. 4a,b), the analysis of the KU55933-treated keratinocytes displayed strikingly decreased K1 and K10 expression and increased Involucrin expression (Supplementary Fig. 4c). Consistent with our observation, it has been shown that normal human skin is characterized by a more prominent pATM/ATM staining in the basal layers of the epidermis, indicating that ATM expression is down-regulated upon the initiation of epidermal differentiation^45^. The analysis of the KU55933-treated keratinocytes showed that K1 and K10 expression was decreased and that Involucrin expression was increased; this pattern of marker expression was observed at the transition from the spinous to the granular layer. This observation indicates that ATM down-regulation might play a role in the transition from the spinous to the granular layer; however, the exact stage at which ATM is involved in keratinocyte differentiation remains to be clarified.

### *ATP2C1* RNA interference enhances Notch1 expression, promotes keratinocyte differentiation, and suppresses cell migration

**Figure 5:**
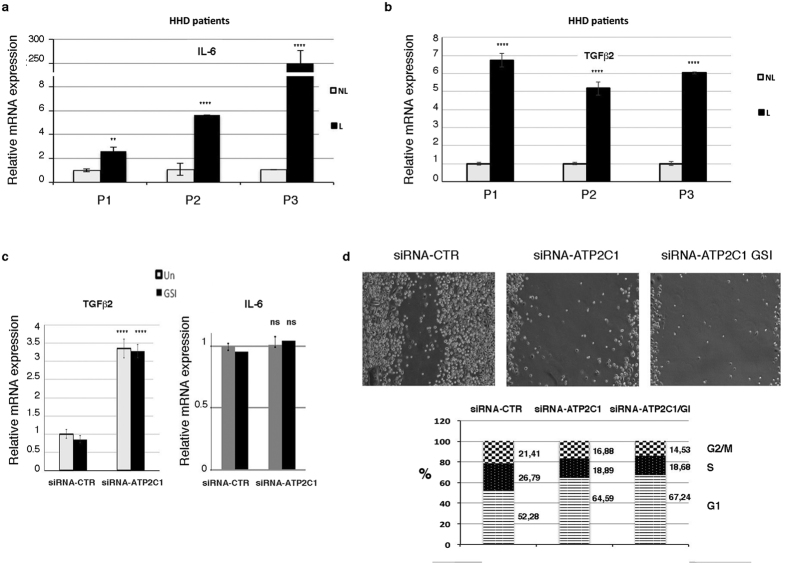
(**a,b**) qRT-PCR analysis of IL-6 and TGF-beta-2 mRNA expression levels in the non-lesioned and lesioned skin of HHD patients. The values are expressed as the fold changes of the indicated samples *vs* the siRNA-CTR-treated, GSI-treated, and untreated samples. (**c**) qRT-PCR of TGF-beta-2/IL-6 in the siRNA-ATP2C1- or siRNA-CTR-transfected NEHKs. (**d**-Upper panel) A scratch assay was performed to assess the migration rates of keratinocytes transfected with the control siRNA-CTR or ATP2C1-specific siRNAs for 48 hrs. Photographs were taken at the indicated time points after scratch injury. Where indicated, the scratch assay was performed in the presence of DMSO as a vehicle control or GSI (10 μΜ), which was added to the cells after the scratch injury (10X magnification). (**d**-Lower panel) Analysis of the cell cycle phases in NHKCs transfected with siRNA-ATP2C1 or siRNA-CTR. Cells stained with propidium iodide were subjected to flow cytometry analysis to determine the cell cycle distribution. The stacked bars represent the mean percentage of cells in a given phase. All graphs are representative of at least three independent experiments, and the values are expressed as the means ± SD of experiments conducted in triplicate. The significance of the differences was calculated using two-tailed Student’s *t* test, with non-lesioned skin samples as the reference. ****P < *0.01*; **P < *0.05*.

HHD is characterized by skin lesions that do not heal and by recurrent skin infections, indicating that HHD keratinocytes might not respond well to challenges such as wounding or infection. After injury to both mouse and human skin, an increase in local cytokine production from keratinocytes occurs^46^,^47^,^48^. In skin wounds, interleukin (IL)-6, the IL-1 family and transforming growth factor (TGF)-beta, crucial cytokines that regulate the re-epithelialization process, are produced locally^49^,^50^,^51^,^52^,^53^,^54^,^55^. We examined whether HHD lesion-derived keratinocytes were defective in wound-induced cytokine production. In our RNA-seq analysis, we found that several factors involved in the wound response were up-regulated, including IL1-θ, TGF-beta2, Toll-like receptor (TLR)2, TLR1, TLR6, IL-6, IL-32, IL-34, and Serum Amyloid A1 and A2. Interestingly, the IL1R2 receptor, which may serve to terminate IL-1-driven inflammation^56^, was down-regulated in HHD lesions. IL-6 and TGF-beta-2 are very important for skin repair. Thus, we further analyzed the expression levels of these cytokines by qRT-PCR. The increases in IL-6 and TGFbeta-2 were confirmed by qRT-PCR in the three patients analyzed (Fig. 5a,b). In primary keratinocytes treated with siRNA-ATP2C1 and the siRNA control, TGF-beta-2 (but not IL-6) expression was increased, indicating that *ATP2C1* loss had a direct effect on TGF-beta-2 expression (Fig. 5c). Therefore, these data indicate that keratinocytes derived from the HHD lesions are not defective in the production of the wound signal but that the poor healing of HHD lesions may result from other aspects of wound repair. Notch signaling controls a number of cellular functions in keratinocytes. Notch down-regulation in the epidermis appears to contribute to tissue regeneration during wound healing^57^. In our analysis, we found that ATP2C1 inhibition resulted in Notch1 activation (Fig. 3), which would alter the wound repair process. Thus, we first analyzed whether ATP2C1 inhibition might affect wound repair using a scratch wound healing assay of primary keratinocyte monolayers. Interestingly, ATP2C1 down-regulation played a role in regulating keratinocyte migration in the scratch wound (Fig. 5d). However, Notch1 inhibition did not rescue the migration properties of keratinocytes in the scratch assay, suggesting that the induction of Notch1 activity by ATP2C1 down-regulation does not play a role in regulating the wound repair of HHD keratinocytes. Additionally, we observed that the scratch wound defect might be the result of compromised proliferation as a consequence of the loss of *ATP2C1* function (Fig. 5d, lower panel). However, the proliferation rate of siRNA-ATP2C1-treated cells was not affected by Notch1 inhibition, indicating that neither the proliferation nor the wound healing of ATP2C1-defective keratinocytes require Notch1.

## Discussion

### Role of *ATP2C1* in human keratinocytes

**Figure 6:**
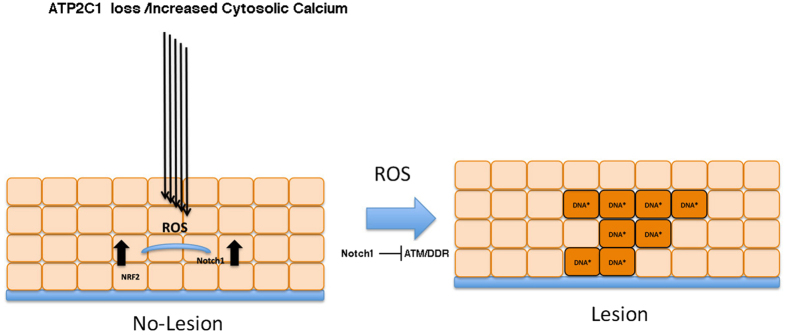
Proposed model of the mechanism involved in the manifestation of Hailey-Hailey disease. The loss of one functional ATP2C1 allele alone is not sufficient to cause clinically overt HHD and skin lesions; additional alterations, e.g., infections or lesions, disrupt the compensatory mechanisms, resulting in deregulated calcium homeostasis. Therefore, our model of the mechanism of HHD pathogenesis is that altered calcium homeostasis produces oxidative stress and subsequent Notch1 activation. Notch1 activation can down-regulate ATM, leading to DNA damage that would then trigger terminal differentiation. However, the shift toward the generation of differentiated cells can irreversibly impact either ESCs or transit amplifying cells, resulting in compromised skin repair.

Previous studies have shown that *ATP2C1* plays an essential role in the maintenance of skin homeostasis, and the loss of *ATP2C*1 function has a causative role in HHD^6^,^7^,^26^,^58^. However, it is unclear how *ATP2C1* loss affects keratinocyte homeostasis. DNA damage is a crucial stage of MYC-mediated replication and stress-induced keratinocyte differentiation^20^, and DNA damage induced by genotoxic agents triggers squamous differentiation^20^,^59^,^60^. In this report, our results show that human keratinocytes respond to the loss of ATP2C1 function in a manner consistent with DNA damage-induced differentiation. This process is paralleled by increased Notch1 activation. Notch signaling is an essential regulatory determinant of keratinocyte growth and differentiation^44^. Human cells expressing Notch1 show inactivation of ATM and other DDR components^19^,^40^. Interestingly, down-regulation of the DDR has been proposed to constitute part of a mechanism associated with astrocyte differentiation^61^. Consistent with this model, it has been shown that the DDR is down-regulated upon the initiation of epidermal differentiation; furthermore, Human Papillomavirus actives the ATM/DNA damage pathway for viral genome amplification upon differentiation^62^. Thus, the loss of *ATP2C1* may allow Notch1 activation to trigger the differentiation response. This response would be augmented by Notch1-mediated inhibition of the DNA repair/ATM pathway in cells that accumulate irreparable DNA damage (Fig. 6). One keratinocyte-specific function of ATP2C1 might be to protect the epidermal cells from a temporally inappropriate activation of Notch1, as HHD is a skin-specific disease.

### Implications for Hailey-Hailey disease

HHD patients exhibit skin blistering from acantholysis, indicating that cell-cell adhesion is compromised in *ATP2C1*-deficient keratinocytes. Given the key role for desmosomes in maintaining tissue integrity and epidermal organization, we analyzed the expression levels of adhesion molecules in the lesioned tissue from HHD patients to clarify the potential involvement of the deregulation of adhesion factors in the acantholysis of HHD lesions. An examination of our RNA-seq data revealed the there was no obvious difference in the adhesion molecule levels in any of the lesions from the analyzed patients. Therefore, although substantial evidence indicates that *ATP2C1* loss compromises adhesion, cellular signaling directly or indirectly regulates desmosomal adhesion. Desmosome adhesion is calcium-independent but reverts to calcium dependence upon wounding in both cultured cell sheets and the epidermis^63^. Remarkably, the mRNA levels of cytokines involved in the wound response were up-regulated in the lesioned skin of HHD patients, indicating that HHD keratinocytes activate repair signaling to maintain skin homeostasis, as shown in other chronic inflammatory diseases^21^,^64^. In particular, the mRNA levels of the proinflammatory cytokines IL-6, IL-1 and IL-32-34 were up-regulated. Among these cytokines, IL-32 produced by keratinocytes has been shown to increase KC apoptosis in inflammatory skin diseases. Interestingly, IL-32 is expressed by human primary keratinocytes and modulates keratinocyte apoptosis in atopic dermatitis, whereas it was not present in either skin biopsy specimens from healthy donors or lesioned skin from patients with psoriasis^65^. Conversely, IL-33 levels are decreased in keratinocytes derived from HHD lesions; IL-33 may play pivotal roles in the maintenance of cutaneous homeostasis and the acceleration of normal wound healing. IL-33 promotes the healing of *Staphylococcus aureus*-infected wounds in mice^66^,^67^; one of the most common causes of skin infections in the HHD lesions is *Staphylococcus aureus*. Together, these findings reveal that keratinocytes derived from the HHD lesions are characterized by deregulated cytokine expression and decreased repair properties. Thus, it is likely that the chronic wounds present in ATP2C1-defective keratinocytes might influence cellular adhesion, possibly through an effect on the intracellular calcium concentrations by shifting cell adhesion from a calcium-independent to a calcium-dependent process.

## Conclusions

Taken together, our findings indicate that *ATP2C1* is involved in skin homeostasis by participating in various cellular processes. The keratinocytes derived from the clinically symptom-less skin of HHD patients behave like keratinocytes derived from healthy donors. We show that ATP2C1 expression and proliferation did not differ significantly between the non-lesion and healthy donor keratinocytes^11^,^12^. However, ATP2C1 expression was reduced in the keratinocytes from the lesioned skin, indicating that an unknown mechanism is responsible for its reduced expression level in the lesioned skin from HHD patients, thus initiating the chain of the molecular events that lead to lesion development. Our hypothesis is that the deregulation of calcium homeostasis resulting from the loss of *ATP2C1* function produces ROS-induced DNA damage. *ATP2C1* loss would then trigger a mechanism that results in Notch1 activation and subsequent ATM down-regulation. Increased ROS levels and ATM loss would produce DNA damage up to a threshold that keratinocytes cannot repair, which would then promote terminal differentiation. HHD is a complex condition characterized by poor wound healing. In the classical model of wound healing, the regenerative capacity of the skin relies on epidermal stem cells (ESCs)^52^. Under normal homeostatic conditions, disruption of epidermal integrity triggers a wound response that induces the recruitment of ESCs to replenish the lost cells. Efficient wound repair relies on the maintenance of both the ESC niche and the production of cells that undergo a limited number of divisions before differentiating as they replenish the damaged tissue. Our hypothesis is that the loss of *ATP2C1* leads to the premature differentiation and exhaustion of the transit amplifying keratinocytes, resulting in compromised skin repair. However, there are still many unanswered questions; it is not clear to what extent the alterations in gene expression observed in the lesioned HHD skin represent a direct response to *ATP2C1* loss and reflect downstream alterations. The discrepancy in Notch1 expression between the lesion-derived and siRNA-ATP2C1-treated keratinocytes in our *in vitro* model may result from the pathways that were altered prior to lesion formation that likely are causative and other alterations in the lesioned area that are likely secondary and associated with tissue damage. However, it might also be possible that these alterations underlie the signaling pathways that are responsible for the initiation and progression of the lesions. A future comparison of the gene expression profiles between lesion-derived and siRNA-ATP2C1-treated keratinocytes may provide more insights into the fundamental biological processes that underlie lesion manifestation.

## Methods

### Patients with Hailey-Hailey disease

This study was conducted according to principles of the Declaration of Helsinki and was approved by the S. Gallicano Institute Ethical Committee. “Written informed consent” was obtained from all subjects. Three familial HHD cases were included in the study. In each patient, diagnosis was established based on the clinical features and the medical and familial histories. Genomic DNA was extracted from whole blood using a Qiagen Blood mini kit (Qiagen, Milan, Italy). All translated *ATP2C1* exons and exon-intron boundaries were amplified using 28 primer pairs^10^. The amplification products were then sequenced in both directions using an automatic sequencing system (310; Applied Biosystems, Foster City, CA, USA). The sequencing results were analyzed with reference to cDNA *ATP2C1* sequence (ENST00000508532). ATP2C1 mutations in these patients, P1, P2 and P3, were previously described^11^.

#### RNA-seq and whole exome sequencing data

The RNA-seq data discussed in this publication have been deposited in the NCBI Gene Expression Omnibus and are accessible through GEO Series accession number GSE77446 (https://www.ncbi.nlm.nih.gov/geo/query/acc.cgi?acc=GSE77466). The Whole Exome sequencing Biosample records are accessible at http://www.ncbi.nlm.nih.gov/biosample/4450420.

## Additional Information

**How to cite this article**: Cialfi, S. *et al*. The loss of ATP2C1 impairs the DNA damage response and induces altered skin homeostasis: Consequences for epidermal biology in Hailey-Hailey disease. *Sci. Rep.* **6**, 31567; doi: 10.1038/srep31567 (2016).

## Supplementary Material

Supplementary Information
