# Corrigendum: The loss of ATP2C1 impairs the DNA damage response and induces altered skin homeostasis: Consequences for epidermal biology in Hailey-Hailey disease

**DOI:** 10.1038/srep44514

**Published:** 2017-03-16

**Authors:** Samantha Cialfi, Loredana Le Pera, Carlo De Blasio, Germano Mariano, Rocco Palermo, Azzurra Zonfrilli, Daniela Uccelletti, Claudio Palleschi, Gianfranco Biolcati, Luca Barbieri, Isabella Screpanti, Claudio Talora

Scientific Reports 6: Article number: 3156710.1038/srep31567; published online: 08 16 2016; updated: 03 16 2017

In this Article, the Gene Expression Omnibus (GEO) accession number for the RNA sequencing data was incorrectly given as GSE77446. The correct accession number is GSE77466.

