# Supplementary material for: The loss of ATP2C1 impairs the DNA damage response and induces altered skin homeostasis: Consequences for epidermal biology in Hailey-Hailey disease
Source: Sci Rep. 2016 Aug 16;6:31567. doi: 10.1038/srep31567 (PMC4985699; doi:10.1038/srep31567)
Supplement: Supplementary Information [file srep31567-s1.pdf]

Revised Supplementary Information

TITLE:

The loss of ATP2C1 impairs the DNA damage response and induces altered skin homeostasis: Consequences for epidermal biology in Hailey-Hailey disease

Authors

Samantha Cialfi[1], Loredana Le Pera[2], Carlo De Blasio[1], Germano Mariano[1], Rocco Palermo[2], Azzurra Zonfrilli[1], Daniela Uccelletti[3], Claudio Palleschi[3], Gianfranco Biolcati[4], Luca Barbieri[4], Isabella Screpanti[1,5] and Claudio Talora[1]*

[1]Department of Molecular Medicine; Sapienza University of Rome; Rome, Italy; [2]Center for Life Nanosciences at Sapienza; Istituto Italiano di Tecnologia; Rome, Italy; [3]Department of Biology and Biotechnology "C. Darwin"; Sapienza University of Rome; Rome, Italy; [4]Porphyria Center, San Gallicano Institute IRCCS, Rome, Italy; [5]Istituto Pasteur, Fondazione Cenci-Bolognetti,

Contact Information: C Talora, Sapienza University of Rome, Viale Regina Elena 291 00161 Rome ITALY E-mail: claudio.talora@uniroma1.it

Additional Title: Molecular mechanism of Hailey-Hailey disease

Page Footnotes: 6 figures; 6 supplementary figures and 1 Table

Supplemental Experimental Procedure

Primary human keratinocytes

Primary human keratinocytes were established from 4-mm punch biopsies derived from healthy donors and patients with HHD as previously described [1]. Cells were maintained in modified low calcium medium (Clonetics; Cambrex Bio Science, Walkersville, MD, U.S.A.). Cells at passages 1 and 2 were used for study purposes.

Cell culture and transfection

NHEK and HaCaT cells (70–80% confluent) were maintained in modified low calcium medium and transfected using the Lipofectamine RNAiMAX transfection Reagent according to manufacturer's instructions (Thermo Fisher Scientific, MA USA). Primary keratinocytes were transfected with 100 nmol L^{-1} small interfering RNAs (siRNAs) for validated human ATP2C1 (L-006119-00; Thermo Scientific/Dharmacon, Lafayette, CO, U.S.A.) and corresponding control scrambled siRNAs Cells were analyzed at the indicated times after transfection by either CM-H2DCFDA analysis for ROS detection or Western blot as indicated [2].

Proliferation assay

Keratinocytes were plated at $3x10^4$ cells/cm^2 and after 24 hours, cells were either untreated or treated with 1mM $CaCl_2$. Proliferation after 72 hours was determined using a cell proliferation reagent (WST-1; Roche-Sigma-Aldrich; Milan, Italy) according to the manufacturer's instructions: WST-1 reagent was added to each well (1/10 dilution) and left for 1 hours in a humidified atmosphere (37°C, 5% CO_2). WST-1 is a tetrazolium salt that, on contact with metabolically active cells is cleaved, and produces formazan dye that can be detected spectrophotometrically using an enzyme-linked immunosorbent assay reader at 450 nm.

Reagents and immunoblotting

The following reagents were purchased from Santa Cruz Biotechnology, Santa Cruz, CA, U.S.A: tubulin, p21Waf1/Cip, Notch1-C20, p53, NRF2. In addition we used ATP2C1 from Abcam (Cambridge, MA, U.S.A.) and Notch1 Val1744, ATM were purchased from Cell Signaling Technology (Beverly, MA, U.S.A). gamma-secretase inhibitor IX (DAPT), was purchased from Calbiochem (Merck KGaA), dissolved in dimethyl sulfoxide (DMSO) and stored at -20 until use. All cell extracts were prepared according to the manufacturer's instructions for detection of phosphor-ERK (Cell Signaling Technology, Beverly, MA, U.S.A.). Quantification of western blots were determined using Image Studio Lite Western Blot Analysis Software Lincoln, NE (USA) and normalized against loading control.

RNA analysis and reverse transcriptase-polymerase chain reaction

Total RNA was isolated from cells, in guanidine isothiocyanate (Trizol reagent, Thermo Fisher Scientific, MA USA)) and further processed by reverse transcriptase-polymerase chain reaction (RT-PCR) as described [3]. Each sample was analysed in triplicated by qRT-PCR and in at least three independent experiments. qRT-PCR was

performed at the opportune annealing temperature with the primers indicated in Table1 with SensiFAST SyBr Hi-ROX kit (Bioline, UK) or with specific TaqMan MGB primers/probe using Taqman gene expression assay (Thermo Fisher Scientific, MA USA).

Reactive oxygen species detection in primary keratinocytes

Primary keratinocytes at passage 2 were cultured at either 60% or 80% confluence. After washing cells in pre-warmed phosphate-buffered saline, culture media were replaced with low calcium medium that had been pre-incubated at 37 °C in a 5% CO_2 atmosphere. Cells were then treated with 5 μmol L^{-1} CM-H2DCFDA (Molecular Probes, Eugene, OR, U.S.A.) for 45 min in the dark, and detached with trypsin–ethylenediamine tetracetic acid solution. After a brief washing, cells were analysed using a FACS flow cytometer (Becton & Dickinson, Franklin Lakes, NJ, U.S.A.).

Next-generation sequencing and data analysis

Whole Exome Sequencing. Genomic DNA was extracted from cultured HHD-cells of HHD-patients using tissue kit (Qiagen, Milano, Italy). Exonic DNA from lesion skin of two HHD patients was targeted by the Sure Select Human All Exon V5 Kit (Agilent) and was sequenced in paired-end (2x100 bp reads) on an Illumina Hiseq2500 platform, with chemistry v3.0. Exome sequencing produced 100,518,042 and 84,100,315 paired-end reads respectively. The Fastq file of each sample was analyzed using the software tool FASTQC v0.10.1 (http://www.bioinformatics.babraham.ac.uk/projects/fastqc/), which provides information about the sequencing quality (data not shown). Sequenced reads were mapped to the human genome build GRCh37 (hg19) using BWA v0.6.2 [4] and the BAM files were sorted [5] using Picard v1.119 (http://broadinstitute.github.io/picard/), duplicate reads were marked before feeding the BAM files into GATK v1.5 [6]. After realignment and mapping quality recalibration, the variants were detected with UnifiedGenotyper, then they were annotated with the tools SNPeff v2.0.5 [7] and PICMI [8], which predict the effects of variants on the genes (such as amino acid changes in the corresponding proteins). Variants mapping onto the target exome region (50.4 Mb) were used for further analysis, with a total of about 43,000 single nucleotide variants (SNVs) for each patient (Supplementary Figure 2A). The variants were then filtered out (following GATK recommended best practices) with respect to annotation metrics, such as quality by depth, mapping quality, variant position within reads and strand bias (QD<2.0, MQ>40.0, FS>60.0, MQRankSum<-12.5, ReadPosRankSum <-8.0). To eliminate common germline polymorphisms from consideration, variants that had same position and nucleotide change, as variants present in database dbSNP 142, with minor allele frequency (MAF) >= 0.01 (in at least one of 14 major populations) and not known to cause clinical phenotypes, were excluded; this filter narrowed the list of variations that might warrant further evaluation for clinical signficance from about 40,000 to about 5,000 single nucleotide variations (Supplementary Figure 2A). A total of about 1,500 putative novel SNVs for each patient were identified removing all the variants already annotated in database dbSNP 142 (without any filter on MAF or other parameters).

RNA-seq. For transcriptome sequencing, total RNA was extracted using the Trizol-Reagent (Life technologies). RNA-seq libraries were prepared following Illumina TruSeqRNA sample preparation protocol. The samples were sequenced using Illumina Hiseq 2500 instrument and ~50 million 100-nt reads per sample were generated. The Fastq file of each sample was analyzed using the software tool

FASTQC v0.10.1 (data not shown). Mapping of reads to reference genome (hg19) was performed using STAR v 2.3.0e [9]. Only reads with unique mapping positions were considered for read counting. Raw read counts were created using HTSeq [10] with the Ensembl GTF transcriptome. Only reads overlapping exon-features were counted. All reads mapping to features with the same identifier were summed. Hereby, the "gene"-attribute was used as feature identifier. Reads mapping to multiple features with different identifier were ignored for read counting. A Trimmed Mean of M-values (TMM) normalization was performed using the edgeR package[11,12] (Supplementary Figure 5). Only genes with at least one count per million in at least three samples were kept for further analysis. The edgeR package was used to compare expression levels of genes between lesional and non-lesional skin samples and identify differentially expressed genes with FDR<0.05 (Benjamini-Hochberg correction for multiple testing).

Supplemental References

1. Cialfi, S., *et al.* Complex multipathways alterations and oxidative stress are associated with Hailey-Hailey disease. *Br J Dermatol* **162**, 518-526 (2010).
2. Cialfi, S., *et al.* Loss of Notch1-dependent p21(Waf1/Cip1) expression influences the Notch1 outcome in tumorigenesis. *Cell cycle* **13**, 2046-2055 (2014).
3. Vargas Romero, P., *et al.* The deregulated expression of miR-125b in acute myeloid leukemia is dependent on the transcription factor C/EBPalpha. *Leukemia* **29**, 2442-2445 (2015).
4. Li, H. & Durbin, R. Fast and accurate short read alignment with Burrows-Wheeler transform. *Bioinformatics* **25**, 1754-1760 (2009).
5. Li, H., *et al.* The Sequence Alignment/Map format and SAMtools. *Bioinformatics* **25**, 2078-2079 (2009).
6. McKenna, A., *et al.* The Genome Analysis Toolkit: a MapReduce framework for analyzing next-generation DNA sequencing data. *Genome research* **20**, 1297-1303 (2010).
7. Cingolani, P., *et al.* A program for annotating and predicting the effects of single nucleotide polymorphisms, SnpEff: SNPs in the genome of Drosophila melanogaster strain w1118; iso-2; iso-3. *Fly* **6**, 80-92 (2012).
8. Le Pera, L., Marcatili, P. & Tramontano, A. PICMI: mapping point mutations on genomes. *Bioinformatics* **26**, 2904-2905 (2010).
9. Dobin, A., *et al.* STAR: ultrafast universal RNA-seq aligner. *Bioinformatics* **29**, 15-21 (2013).
10. Anders, S., Pyl, P.T. & Huber, W. HTSeq--a Python framework to work with high-throughput sequencing data. *Bioinformatics* **31**, 166-169 (2015).
11. Robinson, M.D., McCarthy, D.J. & Smyth, G.K. edgeR: a Bioconductor package for differential expression analysis of digital gene expression data. *Bioinformatics* **26**, 139-140 (2010).

12. Robinson, M.D. & Oshlack, A. A scaling normalization method for differential expression analysis of RNA-seq data. *Genome biology* **11**, R25 (2010).

Supplemental Figure legend

Supplementary Figure 1. Representative qRT-PCR analysis of gene expression for FoxM1, ERCC1 and CHK1, in non-lesional (N) and lesional-skin (L) of HHD patients. The amount of each mRNA was calculated by comparative Ct method and expressed as fold change ($2^{-\Delta\Delta Ct}$). Values were expressed as percentage of decrease with respect to the non-lesional skin set to 1. All graphs are representative of at least three independent experiments and values are expressed in mean ± SD of experiment conducted in triplicate. Values were calculated by two-tailed student *t* test with Non lesional skin sample as the reference. *** *P<0.001;* * *P* < 0.02;

Supplementary Figure 2.. Variant calling results of whole exome sequencing analysis for two HHD-lesion derived keratinocytes (P4, P5). **a**) The total of single nucleotide variants (SNVs) for each patient and the corresponding results for each analysis filtering step: on the target exome region (50.4 Mb); passing the parameter filters (as described in the Methods); subtracting common alleles (Minor Allele Frequency >=0.01) thought to have no medical impact, as reported in dbSNP (build 142). **b**) Characterization of the identified single nucleotide variants as silent (or synonymous), missense (or non-synonymous), nonsense (or stop-gain), stop-lost, splice site acceptor and splice site donor mutations. In the last columns, the variations in common between the two patients (same genome coordinates and same nucleotide changes) and the corresponding genes harboring them are reported.

Supplementary Figure 3. Variant calling results of whole exome sequencing analysis for two HHD-lesions derived keratinocytes (P4,P5). **a,b**) List of genes with STOP-gain identified in the WES analysis. ATP2C1 mutation for both Patients 4 and 5 is shown. These mutations (described in Cialfi et al., 2010) were also found by WES analysis, at the genomic position chr3:130685987 A>T; chr3:130660452 T>A (GRCh37/hg19) for P4 and P5 respectively.

Supplementary Figure 4. (**a-c**) qRT-PCR analysis of gene expression for the indicated targets in primary keratinocytes treated with DMSO or ATM inhibitor KU55993. Values were expressed as fold change, of decrease with respect to the DMSO treated vs KU55993 treated cells. (**b**) Western blot is shown as control of ATM inhibition after KU55993 treatment. All graphs are representative of at least three independent experiments and values are expressed in mean ± SD of experiment conducted in triplicate. Values were calculated by two-tailed student *t* test with vehicle treated sample as the reference. **** *p<0.0001;* *** *p<0.0002* ** *p* < 0.01.

Supplementary Figure 5. Result of trimmed Mean of M-values (TMM) normalization for each sample, performed using the edgeR package.

Supplementary Figure 6. Proliferation rate of normal human keratinocytes in low and high calcium-containing medium; (**a**) Graphs depict proliferation rated in which cells were plated at $3x10^4$ cells/cm^2 and relative proliferation was assessed by WST assay a 3 days after calcium treatment. Values represent mean + SD three independent experiments. **** *p<0.0001*. (**b**) Morphological changes are shown by phase contrast microscopy in normal human keratinocytes cultured in low calcium (UN) and high calcium medium (1mM).

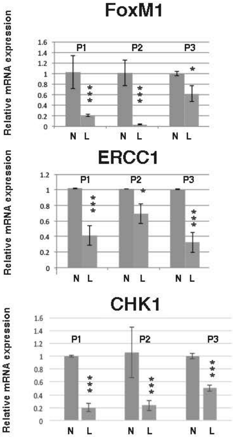
FoxM1
Relative mRNA expression
P1
P2
P3
N L
N L
N L
ERCC1
Relative mRNA expression
P1
P2
P3
N L
N L
N L
CHK1
Relative mRNA expression
P1
P2
P3
N L
N L
N L

a

Single Nucleotide Variants (SNVs)	Patient 4	Patient 5
Total	860467	652745
On target exome region (50.4 Mb)	43835	43300
Passing parameter filters	41515	40992
Excluding the common (MAF>=0.01) variants not known to cause clinical phenotypes	5372	5224

b

Single Nucleotide Variants (SNVs)	Patient 4	Patient 5	Intersection	Genes
Silent	1036	1049	652	470
Missense	2561	2491	1520	954
Nonsense	93	84	45	41
Stop lost	49	47	31	30
Splice site acceptor	11	9	6	6
Splice site donor	9	6	5	5

a

Patient 4

c.1123-2A>T

ABCB6	CROCC	HERC2P3	NFU1	PRIM2	SLC6A18
AC021016.6	CSF2RA	HLA-B	NOC3L	PRUNE2	SRA1
ANKRD36C	CYP2B7P1	HLA-DPB1	NOTCH2NL	RAB17	SYNE2
AP3B1	DOCK10	IDS	NPSR1	RBM3	TCEANC
APOA1BP	DTX2P1	IGSF10	OR4C3	RP11-22B23.1	TNS1
ARHGEF16	EIF2S3	IRS1	OR4C5	RP11-293B20.2	TPTE
ATAD3A	ELAC2	KCNQ1	OR4N3P	RP11-650L12.2	UBE2NL
C9orf174	ERAP1	LAMA4	OR4X2	RP3-368B9.1	VPS33B
CASP12	ESD	MAGEC3	P2RX6	SAMD15	WASF4P
CCDC108	ESRP2	MAP2K3	PAPLN	SCLY	ZDHHC11
CCDC87	FKTN	MLL3	PDE4DIP	SEC22B	ZNF574
CD24P4	FRG1	MLPH	PJA1	SKA3	ZNF583
CHRNA3	GABRA6	MST1P2	PLCD4	SLC25A43	
CNTN5	GYG1	MUC3A	POGZ	SLC29A2	
CPZ	HDAC4	NEURL4	POMT1	SLC2A8	

b

Patient 5

c.440T>A (STOP Gain)

AC021016.6	COMT	HLA-A	OR4C3	RP3-368B9.1	VNN2
AC079612.1	CROCC	HLA-DPB1	OR4C5	SCGB3A2	ZDHHC11
AP3B1	DIS3L2	IDS	OR4N3P	SEC22B	
ARSD	DTX2P1	KCNQ1	PAPLN	SIGLEC10	
ATP2C1	EFCAB10	LAMA4	PDE4DIP	SKA3	
AXDND1	EIF2S3	LSS	PDXDC1	SMPD4	
BRD2	ELAC2	MAK16	PLCD4	SP110	
C2orf83	FAM182A	MAP2K3	PPEF1	SPPL2A	
CARS2	FASTKD2	MLL3	PRIM2	SRA1	
CASP12	FRG1	MUC3A	PTBP1	TCEANC	
CCDC108	GABRA6	NDRG4	RBM3	TMEM176A	
CCDC87	GPC1	NEURL4	RHPN2	TNS1	
CHRNA3	HABP2	NGEF	RP11-293B20.2	TPCN1	
CMAS	HDAC4	NRD1	RP11-397M16.4	UBE2NL	
CNTN5	HERC2P3	OBSL1	RP11-650L12.2	UBXN6	

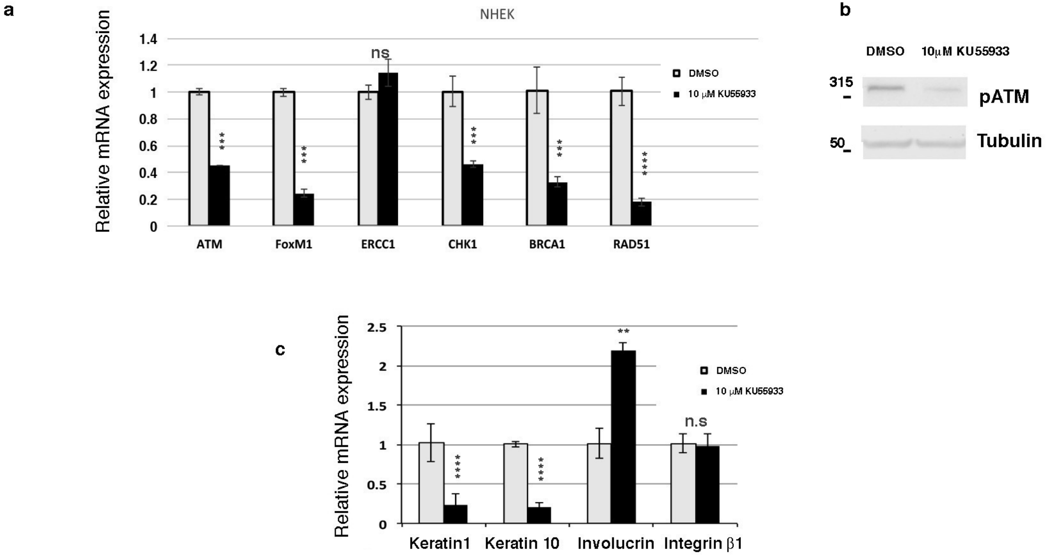
a
NHEK
Relative mRNA expression
1.4
1.2
1
0.8
0.6
0.4
0.2
0
ns
***
***
***
***
****
DMSO
10 μM KU55933
ATM
FoxM1
ERCC1
CHK1
BRCA1
RAD51
b
DMSO
10μM KU55933
315
pATM
50
Tubulin
c
Relative mRNA expression
2.5
2
1.5
1
0.5
0
**
n.s
****
****
DMSO
10 μM KU55933
Keratin1
Keratin 10
Involucrin
Integrin β1

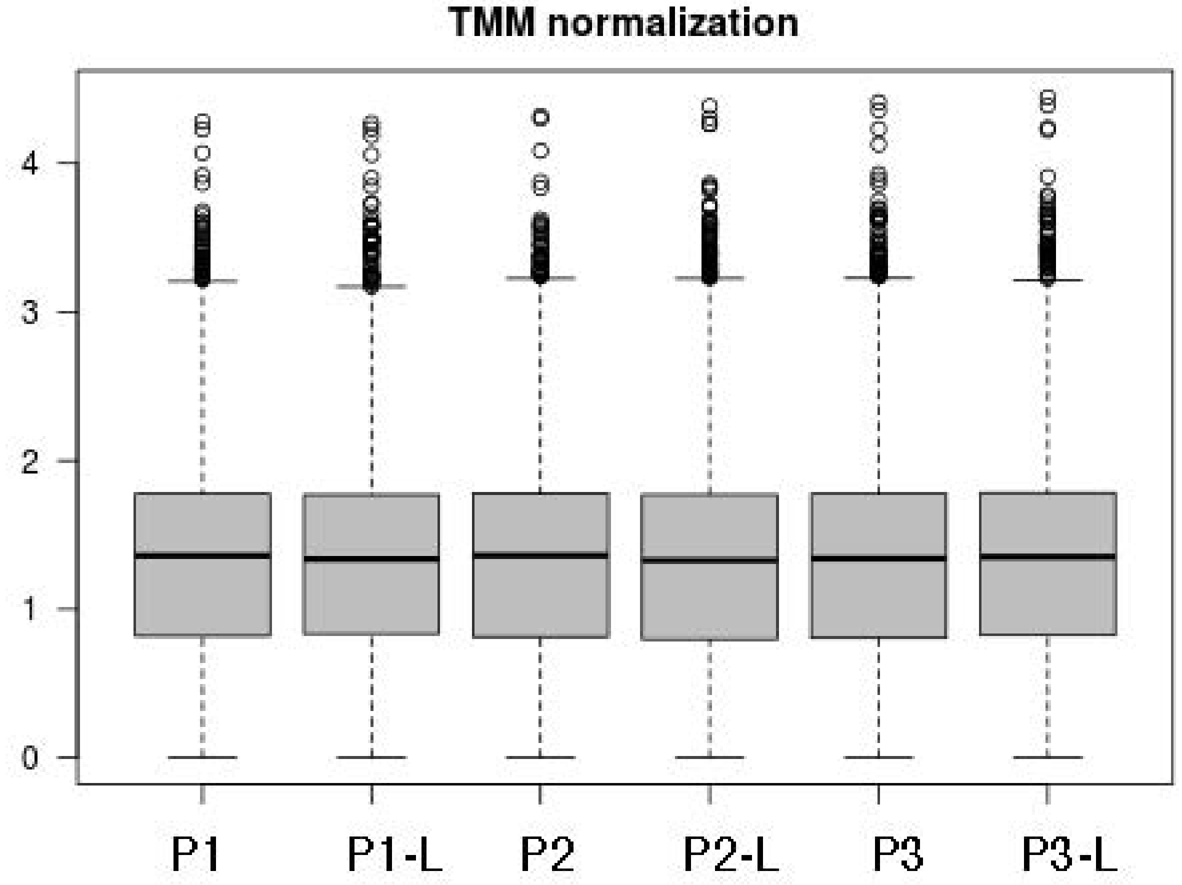

TMM normalization
0
1
2
3
4
P1
P1-L
P2
P2-L
P3
P3-L

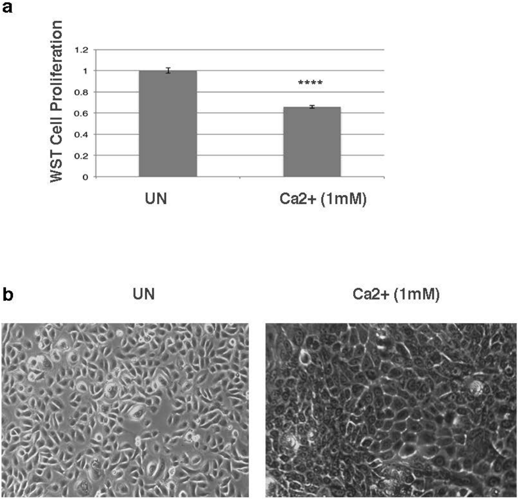
a
WST Cell Proliferation
1.2
1
0.8
0.6
0.4
0.2
0
****
UN
Ca2+ (1mM)
b
UN
Ca2+ (1mM)
